# Increased expressions of CD123, CD63, CD203c, and Fc epsilon receptor I on blood leukocytes of allergic asthma

**DOI:** 10.3389/fmolb.2022.907092

**Published:** 2022-08-11

**Authors:** Hua Xie, Liping Chen, Huiyun Zhang, Junling Wang, Yanyan Zang, Mengmeng Zhan, Fangqiu Gu, Shunlan Wang, Shaoheng He

**Affiliations:** ^1^ The PLA Center of Respiratory and Allergic Disease Diagnosing Management, General Hospital of Northern Theater Command, Shenyang, China; ^2^ Allergy and Clinical Immunology Research Centre, The First Affiliated Hospital of Jinzhou Medical University, Jinzhou, China; ^3^ Translational Medicine Institute, Shenyang Medical College, Shenyang, China; ^4^ Department of Respiratory and Critical Care Medicine, The Second Hospital of Senyang Medical College, Shenyang, China; ^5^ Central Laboratory, Affiliated Haikou Hospital of Xiangya Medical College, Central South University, Haikou, China

**Keywords:** granulocyte, CD63, CD203c, CD123, FcεRI, basophil

## Abstract

**Background** **:** Altered basophil identification markers have been discovered to associate with allergic asthma (AA) in recent years. However, little is known about the expression of basophil markers in blood granulocytes.

**Aim:** To parallel test blood basophils in peripheral blood mononuclear cell (PBMC) and granulocyte populations of patients with AA and AA combined with allergic rhinitis (ARA)

**Methods:** The expressions of surface molecules were determined *via* flow cytometry. CD123 expressing cells in blood were isolated using a cell sorting technique, and mouse AA models were employed for *in vivo* study.

**Results:** The numbers of CD123^+^HLA-DR^−^ cells in the granulocytes of AA and ARA patients markedly increased. However, only 49.7% of CD123^+^HLA-DR^−^ cells in granulocytes and 99.0% of CD123^+^HLA-DR^−^ cells in PBMCs were basophils. Almost all CD123^+^HLA-DR^−^ cells expressed CD63 regardless in granulocytes or PBMC. The numbers of CD63, Fc epsilon receptor I (FcεRI), and CD203c expressing cells markedly enhanced in CD123^+^HLA-DR^−^ granulocytes of AA and ARA patients. Mean fluorescence intensity (MFI) of CD63 and CD203c expressions on CD123^+^HLA-DR^−^ PBMC and granulocytes of AA and ARA patients dramatically elevated. House dust mite extract (HDME) and *Artemisia sieversiana* wild allergen extract (ASWE) enhanced the numbers of CD63^+^CD123^+^HLA-DR^−^ granulocytes and PBMC and the MFI of CD203c expression on CD123^+^HLA-DR^−^ granulocyte of AA and ARA patients. Histamine, tryptase, and PGD2 enhanced proportions of CD123^+^ KU812 cells. ASWE- and HDME-induced AA mice showed upregulated CD63 expression on basophils. In conclusion, upregulated expressions of CD123, CD203c, CD63, and FcεRIα in PBMC and granulocytes of patients with AA and ARA suggest that CD123^+^HLA-DR^−^ cells may contribute to the development of AA and ARA.

## Introduction

Allergic asthma (AA) is a major phenotype of asthma, which often commences in childhood and is associated with a family history of allergic disease (Fuchs et al., 2017). Basophils are known as primary effector cells of allergy (Kepley, 2006), which greatly influence the behavior of eosinophils (Nakashima et al., 2014) and neutrophils (Jonsson et al., 2019). The increased numbers of basophils in the sputum (Brooks et al., 2017) and in baseline biopsy specimens (Macfarlane et al., 2000) of the patients with AA suggest that basophils may play a role in AA.

Over the years, CD13, CD44, CD54, CD63, CD69, CD107a, CD123, CD164, CCR3, CD203c, TLR-4, and FcεRI have been recognized as identification markers of basophils (He et al., 2022). The newly emerging markers of subpopulations of basophils, like CD16b, have also been reported (Heneberg et al., 2019). Some of these markers such as CD203c and CD63 (Abuaf et al., 2008; Imamura et al., 2021) are upregulated upon activation. However, eosinophils also express CD123 (Toba et al., 1999), and neutrophils express CD123 (Smith et al., 1995) in human peripheral blood. In AA, different results have been produced from various laboratories in recent years. For example, decreased CD123^+^ basophils were found in the blood after segmental allergen challenge of the asthmatic lung (Dijkstra et al., 2014), but increased CD203c^+^ basophils were observed in the blood from patients with asthma exacerbation (Ono et al., 2010). Since basophils were recognized in peripheral blood mononuclear cell (PBMC) *via* flow cytometry examination (Zhong et al., 2021) and some of the basophils are multinucleated cells and were originally discovered as one of the granulocytes in mammals (Siracusa et al., 2013), we attempted to parallel examine basophils in PBMC and granulocyte populations of peripheral blood in the current study. Allergen challenge to the airways of atopic asthmatic individuals increases the levels of mast cells and basophils in sputum, and the number of sputum eosinophils and basophils (Gauvreau et al., 2000). Allergen inhalation has been found to elevate the number of basophils in bronchial biopsy specimens (Macfarlane et al., 2000). These studies suggest that allergens may be able to alter basophil behavior under allergic conditions.

The contribution of proinflammatory mediators to the pathogenesis of AA has long been recognized. It was reported that children with asthma had higher serum tryptase levels when compared with healthy controls (Gao et al., 2016), and serum tryptase levels in adult subjects are associated with asthma (Pejler, 2019). Although the levels of prostaglandin D2 (PGD2) and leukotriene C4 (LTC4) were significantly higher in asthmatics than in healthy children (Glowacka et al., 2013), the concentration of histamine in bronchoalveolar lavage fluid (BALF) was significantly higher in atopic asthma patients (Mitsunobu et al., 1998). Allergen challenge in atopic asthmatics resulted in significant increases in histamine and tryptase concentrations in BALF (Wenzel et al., 1988). However, the influence of these proinflammatory mediators on basophils remains largely unknown. The present study aims to examine the expressions of CD123, CD203c, CD63, and FcεRIα in PBMC and granulocytes of patients with AA and ARA and the influence of allergens and proinflammatory mediators on their expression.

## Materials and methods

### Reagents

The following reagents were purchased from Biolegend (San Diego, United States): FITC-conjugated mouse antihuman CD123 antibody (Ab) (Clone: 6H6) and its isotype Ab FITC-conjugated mouse IgG1 κ (MOPC-21), APC/Cy7-conjugated mouse antihuman HLA-DRα Ab (L243) and APC/Cy7-conjugated mouse IgG2a κ (MOPC-173), PerCP-conjugated mouse antihuman FcεRIα Ab [AER-37 (CRA-1)] and PerCP-conjugated mouse IgG2b κ (MPC-11), PE-conjugated mouse antihuman CD203c Ab (NP4D6) and PE-conjugated mouse IgG1 κ (MOPC-21), PE/Cy7-conjugated mouse antihuman CD63 Ab (H5C6) and PE/Cy7-conjugated mouse IgG1 κ (MOPC-21), PE/Cy7-conjugated Armenian hamster antimouse CD49b Ab (HMα2) and PE/Cy7-conjugated Armenian hamster IgG (HTK888), PE-conjugated rat antimouse CD123 Ab (5B11) and PE-conjugated rat IgG2a κ (RTK2758), APC-conjugated rat antimouse CD63 Ab (NVG-2) and APC-conjugated rat IgG2a κ (R35-95), rat antimouse CD16/32 Ab (93), Zombie NIR™ and Zombie Aqua™ Fixable Viability kit, and human Fc receptor-blocking solution and human red blood cell lysis buffer. Wright Giemsa stain was supplied by Baso diagnostics, Inc. (Zhuhai, China). Calcium ionophore A2378 (CI), histamine, DNase I, and Trypan blue dye were obtained from Sigma-Aldrich (St Louis, MO, United States). Alhydrogel^®^ adjuvant was bought from InvivoGen (San Diego, CA, United States). PGD2 Skin rhβ tryptase was from Promega Corporation (Madison, WI, United States). Montelukast sodium, olopatadine hydrochloride, and BAY-u 3405 ramatroban were bought from Tocris Bioscience (Minneapolis, MN, United States). LTC4 was supplied by Abcam (Cambridge, MA, United States). ATCC-formulated RPMI 1640 medium (ATCC30-2001) was purchased from ATCC (Manassas, VA, United States). Red blood cell lysis buffer (Multi-species) for mouse experiment was bought from Invitrogen (Carlsbad, CA, United States). HDME and ASWE were obtained from Macro Union Pharmaceutical Co. Ltd. (Beijing, China). Fetal bovine serum (FBS) (endotoxin content less than 10 IU/ml) and RPMI 1640 medium were purchased from PAN™ Seratech (Aidenbach, Germany) and Corning (NY, United States), respectively. Allergens for skin prick tests were supplied by ALK-Abelló, Inc. (Denmark). Most of the general-purpose chemicals, such as salts and buffer components were of analytical grade.

### Subjects, animals, and cell lines

As presented in Table 1, a total of 125 AA patients, 56 ARA patients, and 52 healthy subjects (HC) were included in the study. AA diagnosis was in accordance with the criteria of the Global Initiative for Asthma (Mauer and Taliercio, 2020). ARA was diagnosed based on allergic rhinitis and its impact on asthma (Hoyte and Nelson, 2018). The study was conducted in compliance with the Declaration of Helsinki and was approved by the Ethical Committees of the First Affiliated Hospital of Jinzhou Medical University and the General Hospital of Northern Theater Command. After obtaining written informed consent in the outpatient clinic, approximately 10 ml of peripheral blood from each patient was taken into a K_2_EDTA-containing tube and centrifuged at 450 g for 10 min. Resultant cells and plasma were collected separately for flow cytometry and ELISA analyses.

**TABLE 1:** General characteristics of patients and HC.

Population	Case	Age (y)	Female/male	History (y)	Onset age (y)
HC	52	30 (12–63)	32/20	0	NA
AA^−^	82	48 (6–75)	54/28	3 (0.25–30)	38 (1–74.75)
AA^+^	43	43 (7–74)	24/19	4 (0.25–30)	34 (2-70)
ARA^−^	19	52 (29–70)	13/6	7 (0.5–26)	38 (18-58)
ARA^+^	37	35 (5–63)	15/22	5 (0.5–33)	30 (3-55)

Notes: Median values (range) are shown. Specific allergens were examined by skin prick test. NA, not applicable; HC, healthy control; AA^−^ = asthma for negative skin prick test; AA^+^ = asthma for positive skin prick test; ARA^−^ = combined allergic rhinitis and asthma syndrome for negative skin prick test; ARA^+^ = combined allergic rhinitis and asthma syndrome for positive skin prick test.

C57BL/6J mice, weighting 18–22 g, were purchased from Vital River Laboratory Animal Technology Co., Ltd. (Beijing, China), and maintained in the First Affiliated Hospital of Jinzhou Medical University under specific pathogen-free facilities with free access to standard rodent chow and water, at a constant temperature 23–28°C and relative humidity of 60%–75%. The animal experiment procedures were authorized by the Animal Care Committee at Jinzhou Medical University.

FcεRIα knockout (KO) mice on C57BL/6J background were developed by GemPharmatech Co. Ltd. (Jiangsu, China) using the CRISPR-Cas9 system (Zhang et al., 2016). In brief, Cas9 mRNA and single guide (sg) RNAs (listed in Table 2)-targeting the introns on both sides of exon 1–5 of FcεRIα (Ensembl no.:ENSMUST00000049706.10) were constructed and transcribed *in vitro*, then microinjected into zygotes of C57BL/6J mice, and further transplanted into the oviduct of pseudopregnant mothers. All mice were genotyped 1 week after birth using PCR with specific primers (shown in Table 3) and sequencing. Generated F0 mice heterozygous for the FcεRIα mutation were then backcrossed with wild-type (WT) mice to breed an F1 heterozygous generation with stable genotypes. F1 generation mice were further crossed to heterozygous siblings to obtain homozygous KO offsprings (shown in Supplementary Figure S1) and used for further study.

**TABLE 2:** Primer sequence information on small guide (sg)RNA and protospacer adjacent motif (PAM).

sgRNA forward primers (5’→3’)	PAM
TGA​TGG​AAA​GCA​ATA​CTA​TC	TGG
ACT​TAA​AAC​ACA​GAG​TAT​AG	AGG
CCT​AGA​AAA​GTC​TGT​TCC​AC	AGG
GTT​ACA​TAA​CCA​GCT​GCA​GT	TGG

**TABLE 3:** Primer sequence information on Fcer1a KO and WT mice.

Name	Primer sequence	Product (bp)
Fcer1a-KO	GGA​GTG​TTT​GAC​TTC​TGC​CCT​G (F)	KO: 742
	GCT​TCT​AGC​AAC​AGA​AGG​CAG​ATT​AC (R)	WT: 6951
Fcer1a-WT	GTG​CCC​TAT​CTT​GAT​GCC​TAT​ATT​GG (F)	KO: 0
	TCC​CAT​ATC​CCT​GGA​ACG​TAC​C (R)	WT: 488

KU812 cells (ATCC^®^ CRL-2099™) were maintained in ATCC-formulated RPMI 1640 base medium supplemented with 10% heat-inactivated FBS and 100 units/ml penicillin/streptomycin in 75-cm^2^ tissue culture flasks at 37°C in a 5% (v/v) CO_2_, water-saturated atmosphere.

### Flow cytometric cell sorting

Fresh peripheral blood samples were first incubated with human Fc receptor-blocking solution for 10 min and then stained with FITC-conjugated antihuman CD123 and APC/Cy7-conjugated antihuman HLA-DRα Abs. Target cells in granulocytes and PBMC were sorted into individual tubes containing RPMI 1640 medium supplemented with 3% FBS on a three-laser SH800 sorter (Sony Biotechnology) in purity mode.

### Preparation of cytospin slides and cell count

Cytocentrifuge slides were prepared with Shanton cytospin 4 (Thermo Fisher Scientific) and stained with Wright Giemsa stain according to the manufacturers’ instructions. Based on the morphology of leukocytes, differential cell counts under the microscope were performed with a minimum count of 100 cells. The results were expressed as the relative percentage of each cell type out of the total cells counted per preparation.

### Gating strategies for human basophil subpopulations in peripheral blood granulocytes and peripheral blood mononuclear cell

Gating strategy for CD123^+^HLA-DR^−^ cells of granulocytes and PBMC was demonstrated in Supplementary Figure S2. In brief, leukocytes were recognized following dead cells and doublets being discriminated by SSC-A-Zombie Aqua and FSC-H-FSC-A gating strategies. Granulocytes and PBMC were then separated from cell debris according to high SSC-A (SSC-A^high^ FSC-A) and low SSC-A (SSC-A^low^ FSC-A), respectively. Cells within the granulocyte population gate and PBMC gate were identified as CD123^+^HLA-DR^−^ cells.

### Flow cytometric analysis of CD123^+^HLA-DR^−^ cells and CD63, CD203c, and FcεRIa expressions

The detection of CD63, CD203c, and FcεRI expressions on human blood CD123^+^HLA-DR^−^ cells in both granulocytes and PBMC was performed as described previously (Zheng et al., 2016) with mild modification. In brief, whole blood cells were incubated in the presence or absence of ASWE or HDME (all at concentrations of 0.1 and 1.0 μg/ml) for 30 min (Sainte-Laudy et al., 2007; Sonneck et al., 2008) at 37°C in a 5% (v/v) CO_2_, water-saturated atmosphere. Cells were subsequently preincubated with human Fc receptor-blocking solution (Cronin et al., 2020) and Zombie Aqua dye (Cossarizza et al., 2019) for 15 min in the dark at room temperature. Each labeled monoclonal antibody (mAb), including FITC-CD123, APC/Cy7-HLA-DR, PerCP-FcεRIα, PE-CD203c, and PE/Cy7-CD63, was added into the tube for 15 min. Following lysis of red blood cells, white blood cells were fixed and permeabilized by using Cytofix/Cytoperm™ Fixation/Permeabilization Kit according to the manufacturer’s instructions. Cells were analyzed with FACS Verse flow cytometer (BD Biosciences). A total of 10,000 events in the live cell gate were analyzed using FlowJo software version 7.0 (Treestar) for each sample. Each irrelevant isotype- and concentration-matched Ab was used for fluorescence minus one (FMO) control.

To detect CD63 expression in mouse blood basophils, whole blood cells were preincubated with antimouse CD16/32 Ab and Zombie NIR dye (Headley et al., 2016). Each labeled Ab including PE-CD123, PE/Cy7-CD49b, and APC-CD63 was added into tubes before lysing erythrocytes. Cells were then processed for human blood samples and analyzed using flow cytometry as described above.

### Flow cytometric analysis of expressions of CD123, CD63, CD203c, and FcεRIa on KU812 cells

The challenging procedure for KU812 cells was mainly adopted from a method previously described by Zhang et al. (2010) for P815 cells. In brief, cultured KU812 cells at a density of 0.5 × 10^6^ cells/ml were incubated with tryptase, histamine (1.0 μg/ml) with or without olopatadine hydrochloride (10 μg/ml), PGD2 (0.1 μg/ml) with or without BAY-u 3405 ramatroban (0.35 μg/ml), LTC4 (300 nM) with or without montelukast sodium (900 nM), CI (1.0 μM) or PBS (pH 7.4) for 30 min, and 2 h or 16 h at 37°C. Cells were then harvested and centrifuged at 300 g for 8 min at 4°C before the culture supernatant was collected and frozen at −80°C. Cell pellets were resuspended for flow cytometry. The challenge tests were repeated four times. For those blocking compounds, they were preincubation with the corresponding reagents for 30 min at room temperature before adding to cells. Cells were analyzed using flow cytometry as described above.

### Establishment of mouse allergic asthma model induced by *Artemisia sieversiana* wild allergen extract and house dust mite extract

ASWE- and HDME-induced AA mouse models were adopted mainly from the models described by Boldogh et al. (2005) and Ryu et al. (2013). In brief, ASWE- and HDME-induced AA mice were sensitized by intraperitoneally injecting 150 μg of ASWE and 100 µg of HDME emulsified in 2 mg of alhydrogel weekly for 3 weeks, respectively. One week after the last sensitization, mice were exposed to a 30 μg/ml of ASWE or HDME aerosol challenge for eight consecutive days. For control experiments, mice received vehicle alone instead of an allergen solution. At 24 h following the last challenge, blood samples were collected from each mouse and were used for flow cytometry analysis.

### Statistics

Statistical analyses were performed by using SPSS version 13.0 software (SPSS, Inc. Chicago, IL, United States). Human peripheral blood data and data for blood granulocyte differential cell counts were displayed as scatter plots, where Kruskal–Wallis analysis indicated significant differences between groups, for the preplanned comparisons of interest, the paired Mann–Whitney U test (MWUT) was employed. Data from KU812 cell lines were presented as mean ± SEM and analyzed using Student’s *t*-test (*t*-test). Data for blood PBMC differential cell counts and animal study were displayed as boxplots, which indicate the median, interquartile range, and the largest and smallest values for the number of experiments indicated. The normality of distribution was determined using the Kolmogorov–Smirnov or Shapiro–Wilk tests (Diana et al., 2018). Correlations were determined using Pearson’s correlation analysis. Correlation coefficient ranges were defined as follows: R < 0.3 as a weak correlation, 0.3 ≤ R ≤ 0.7 as a moderate correlation, and >0.7 as a strong correlation (Lee et al., 2013). For all analyses, *p* < 0.05 was taken as significant.

## Results

### Gating strategies for CD123^+^HLA-DR^−^ cells in both granulocyte and peripheral blood mononuclear cell populations

**FIGURE 1:**
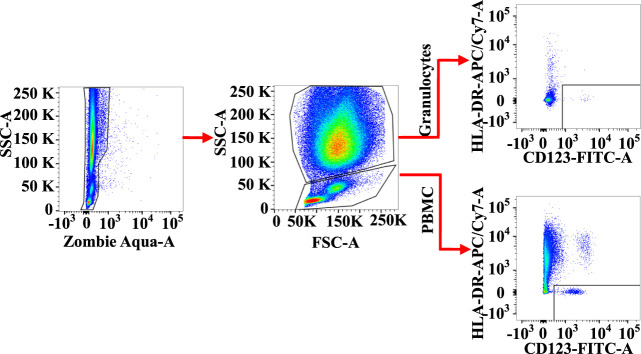
Gating strategies for CD123+HLA-DR− cells in both granulocyte and peripheral blood mononuclear cell (PBMC) populations.

Granulocytes and PBMC in human blood leukocytes were defined according to high SSC-A and low SSC-A via flow cytometry, respectively (Ost et al., 1998). CD123^+^HLA-DR^−^ cells were located in the lower right corner of the figure (Figure 1).

### Altered proportions and numbers of CD123^+^HLA-DR^−^ cells in granulocytes and peripheral blood mononuclear cell of allergic asthma and allergic rhinitis

**FIGURE 2:**
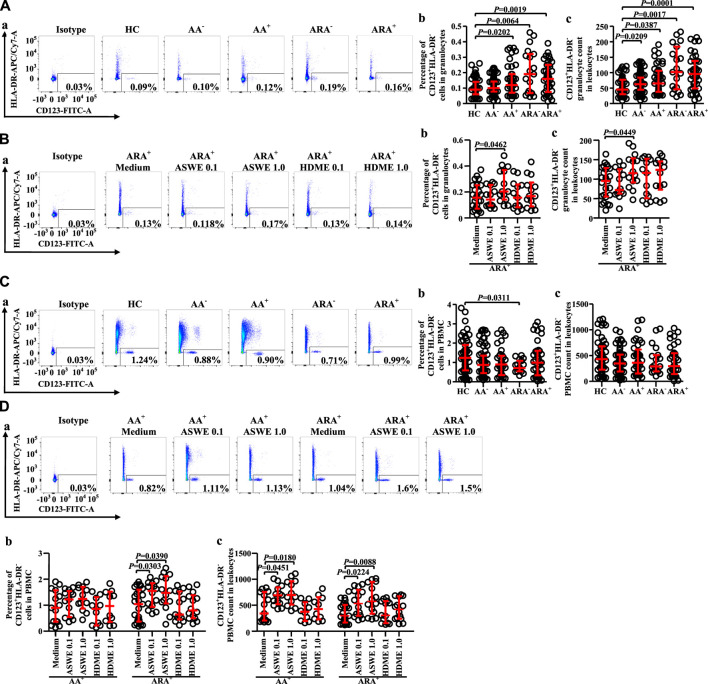
Flow cytometry analysis of proportions and numbers of CD123+HLA-DR− cells in granulocytes and PBMC of allergic asthma (AA) and healthy control (HC) subjects. **(A)** and **(C)** Proportions and numbers of CD123+HLA-DR− cells in granulocytes and PBMC. **(B)** and **(D)** proportions and numbers of CD123+HLA-DR− cells in granulocytes and PBMC after house dust mite extract (HDME, μg/ml) and *Artemisia sieversiana* wild allergen extract (ASWE, μg/ml) challenge. **(a)** representative graphs of the gating strategies. **(b)** Percentages indicated cells of skin prick test (SPT)-negative AA (AA−), SPT-positive AA (AA+), SPT-negative AA combined with allergic rhinitis (ARA−), SPT-positive AA combined with allergic rhinitis (ARA+) patients, and HC subjects. **(c)** numbers of CD123+HLA-DR− cells in leukocytes.

Since basophils are primary effector cells in AA, and little is known of the changes in proportions and numbers of CD123^+^HLA-DR^−^ cells in granulocytes and PBMC of AA and ARA, we investigated the expression of CD123^+^HLA-DR^−^ cells in peripheral blood of AA and ARA. The results showed that the percentages of CD123^+^HLA-DR^−^ cells in granulocytes of AA^+^, ARA^−^, and ARA^+^ patients (Figure 2Aa, b MWUT, *p* = 0.0202, 0.0064, 0.0019) and numbers of CD123^+^HLA-DR^−^ granulocytes in leukocytes of AA^−^, AA^+^, ARA^−^, and ARA^+^ patients (Figure 2Ac, MWUT, *p* = 0.0209, 0.0387, 0.0017, 0.0001) markedly increased. Allergen extract ASWE at 1.0 μg/ml enhanced percentage of CD123^+^HLA-DR^−^ cells in granulocytes (Figure 2Ba, b. MWUT, *p* = 0.0462) and number of CD123^+^HLA-DR^−^ granulocytes in leukocytes of ARA^+^ patients (Figure 2Bc. MWUT, *p* = 0.0449). By contrast, the proportion of CD123^+^HLA-DR^−^ cells decreased in PBMC of ARA^−^ patients (Figure 2Ca, b MWUT, *p* = 0.0311). ASWE elevated the proportion of CD123^+^HLA-DR^−^ cells in PBMC of ARA^+^ patients (Figure 2Da, b, MWUT, *p* = 0.0303, 0.0390), and numbers of CD123^+^HLA-DR^−^ PBMC in leukocytes of AA^+^ and ARA^+^ patients (Figure 2Dc, MWUT, *p* = 0.0451, 0.0180, 0.0224, 0.0088). The MFI of CD123 expression on CD123^+^HLA-DR^−^ granulocytes and PBMC had little change in the blood of AA and ARA patients (data not shown).

### Identification of isolated CD123^+^HLA-DR^−^ cells in peripheral blood granulocytes and peripheral blood mononuclear cell

**FIGURE 3:**
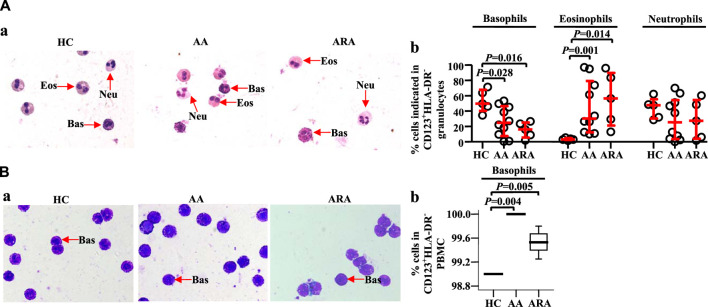
Identification of isolated CD123+HLA-DR− granulocytes and PBMC from human peripheral blood. CD123+HLA-DR− cells in granulocytes **(A)** and PBMC **(B)** were sorted by flow cytometer, and cytocentrifuge slides were prepared. Following staining with Wright Giemsa stain, differential cell counts under a microscope were performed. Basophils have pale blue cytoplasm containing purple-black secondary granules, and the nucleus is segmented. Eosinophils display pink cytoplasm containing large and medium-sized, spherical, dense granules, and segmented nuclei. The cytoplasm of neutrophils is abundant, pale pink containing many small violet-pink neutrophilic or secondary granules distributed evenly throughout the cell with a lobulated nucleus. (A) and (B) representative graphs. (C) and (D) percentages of cells indicated. Data were displayed as a scatter plot for granulocytes and a boxplot for PBMC, which indicates the median, interquartile range, and the largest and smallest values. Each data represented a group of 5–10 separate experiments. Magnification was × 100. The paired Mann–Whitney U test was employed for the analysis of significant differences between groups. For all analyses, *p* < 0.05 was taken as significant by comparison to those obtained with the corresponding HC group.

To confirm the cell types that express CD123 in peripheral blood granulocytes and PBMC, we isolated CD123^+^HLA-DR^−^ cells from human peripheral blood and examined them under a microscope. The results showed approximately 49.7% basophils, 3.0% eosinophils, and 47.7% neutrophils in CD123^+^HLA-DR^−^ granulocytes of HC subjects. AA and ARA patients showed decrease in basophils and increase in eosinophils in CD123^+^HLA-DR^−^ granulocytes (Figure 3Aa, b, MWUT, *p* = 0.028, 0.016, 0.001, 0.014). It is observed that 99.0% (interquartile values P_25_ = 99.0%, P_75_ = 99.0%) CD123^+^HLA-DR^−^ cells in PBMC of HC subjects were basophils (Figure 3Ba). There were more CD123^+^HLA-DR^−^ cells in PBMC of AA and ARA patients than that in HC subjects (Figure 3Ba, MWUT, *p* = 0.004, 0.005).

### Altered expression of CD63 and CD203c in CD123^+^HLA-DR^−^ cell populations of allergic asthma and allergic rhinitis

**FIGURE 4:**
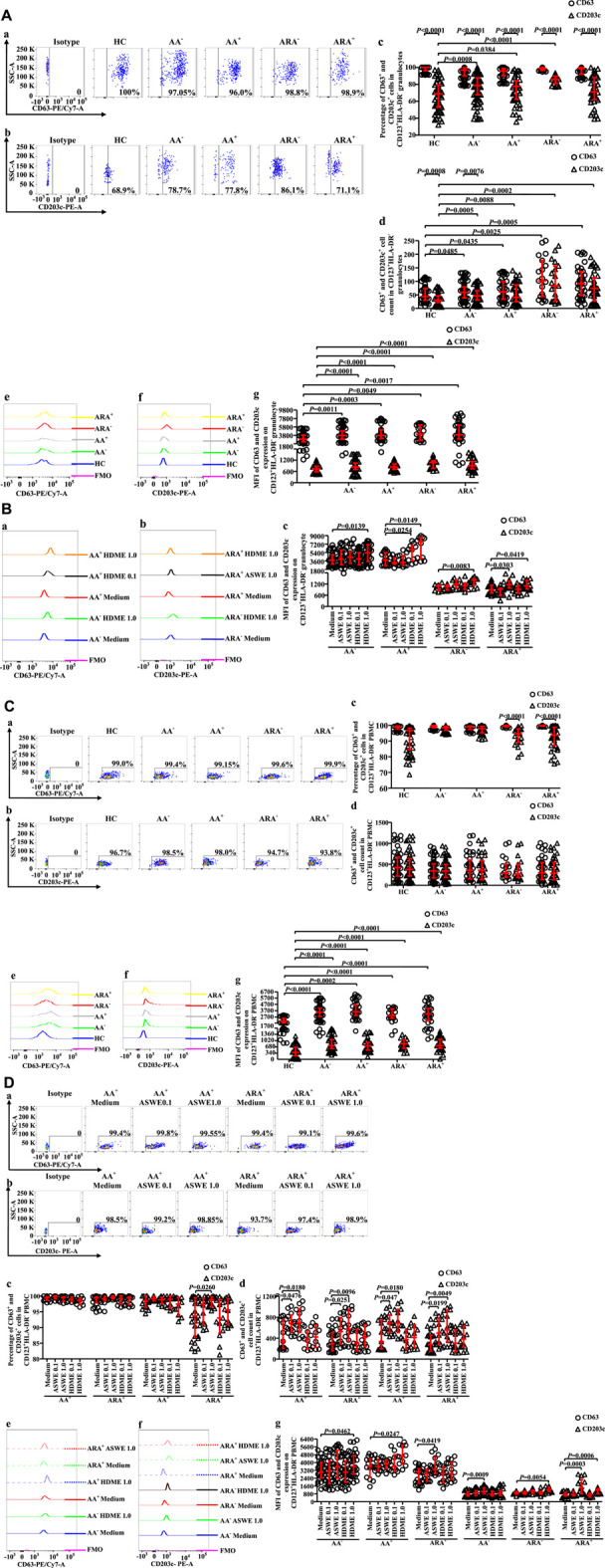
(continued)

CD63 has been considered to be an activation marker of basophils (Abuaf et al., 2008), and increased CD203c^+^ basophils were observed in the blood of patients with asthma exacerbation (Ono et al., 2010). However, little is known about the expressions of CD63 and CD203c on CD123^+^HLA-DR^−^ granulocytes of AA and ARA. We therefore investigated the issue herein. The results showed that almost all CD123^+^HLA-DR^−^ cells expressed CD63 regardless of whether they were in granulocyte (Figure 4Aa, c) or PBMC populations (Figure 4Ca, c) and HC, AA^−^, AA^+^, ARA^−^, and ARA^+^ subjects. However, the proportions of CD203c^+^ cells in CD123^+^HLA-DR^−^ granulocyte (Figure 4Aa, c) and PBMC populations (Figure 4Ca, c) seem less consistent than CD63^+^ cells for HC, AA^−^, AA^+^, ARA^−^, and ARA^+^ subjects. It was observed that the percentages of CD203c^+^CD123^+^HLA-DR^−^ granulocytes increased in AA^−^, AA^+^, and ARA^−^ subjects (Figure 4Aa, c, MWUT, *p* = 0.0008, 0.0384, <0.0001). In comparison with CD63^+^CD123^+^HLA-DR^−^ granulocytes, approximately 19.0%, 19.0%, 12.9%, and 28.1% less CD203c^+^CD123^+^HLA-DR^−^ granulocytes were observed in AA^−^, AA^+^, ARA^−^, and ARA^+^ patients, respectively (Figure 4Aa, b, c, MWUT, *p* < 0.0001, 0.0001, 0.0001, 0.0001). Likewise, 4%.9%, and 6.1% less CD203c^+^CD123^+^HLA-DR^−^ PBMC were found in ARA^−^ and ARA^+^ patients (Figure 4Ca, c, MWUT, *p* < 0.0001, 0.0001). On the other hand, the numbers of CD63^+^CD123^+^HLA-DR^−^ (Figure 4Ad, MWUT, *p* = 0.0485, 0.0435, 0.0025, 0.0005) and CD203c^+^CD123^+^HLA-DR^−^ granulocytes (Figure 4Ac MWUT, *p* = 0.0005, 0.0088, 0.0002, <0.0001), but not the PBMC (Figure 4Cd) in the blood of AA^−^, AA^+^, ARA^−^, and ARA^+^ patients increased.

The MFIs of CD63 and CD203c expressions in CD123^+^HLA-DR^−^ granulocytes (Figure 4Ae, f, g, MWUT, *p* = 0.0011, 0.0003, 0.0049, 0.0017 for CD63; *p* < 0.0001, 0.0001, 0.0001, 0.0001 for CD203c) and PBMC (Figure 4Ce, g, MWUT, *p* < 0.0001, = 0.0002, <0.0001, <0.0001 for CD63; *p* < 0.0001, 0.0001, 0.0001, 0.0001 for CD203c) enhanced in the blood of AA^−^, AA^+^, ARA^−^, and ARA^+^ patients, respectively.

HDME increased the MFIs (Figures 4B,D) of CD63 expression on CD123^+^HLA-DR^−^ granulocytes of AA^−^ and AA^+^ patients (Figure 4Ba, c, MWUT, *p* = 0.0139, 0.0149) and CD203c expression on CD123^+^HLA-DR^−^ granulocytes of ARA^−^ and ARA^+^ patients (Figure 4Bb, c, MWUT, *p* = 0.0083, 0.0419). ASWE elevated the MFIs of CD63 expression on CD123^+^HLA-DR^−^ granulocytes of AA^+^ patients (Figure 4Ba, c, MWUT, *p* = 0.0254) and CD203c expression on CD123^+^HLA-DR^−^ granulocytes of ARA^+^ patients (Figure 4Bb, c, MWUT, *p =* 0.0303). ASWE elevated also the proportion of CD63^+^ cells in CD123^+^HLA-DR^−^ PBMC of ARA^+^ patients (Figure 4Da, c, MWUT, *p* = 0.026), the numbers of CD63^+^CD123^+^HLA-DR^−^ PBMC of AA^+^ and ARA^+^ patients (Figure 4Da, d, MWUT, *p* = 0.0476, 0.0180, 0.0251, 0.0096), and the numbers of CD203c^+^CD123^+^HLA-DR^−^ PBMC of AA^+^ and ARA^+^ patients (Figure 4Db, d, MWUT, *p* = 0.047, 0.018, 0.0199, 0.0049).

### Altered expression of FcεRI on CD123^+^HLA-DR^−^ cell populations of allergic asthma and allergic rhinitis

**FIGURE 5:**
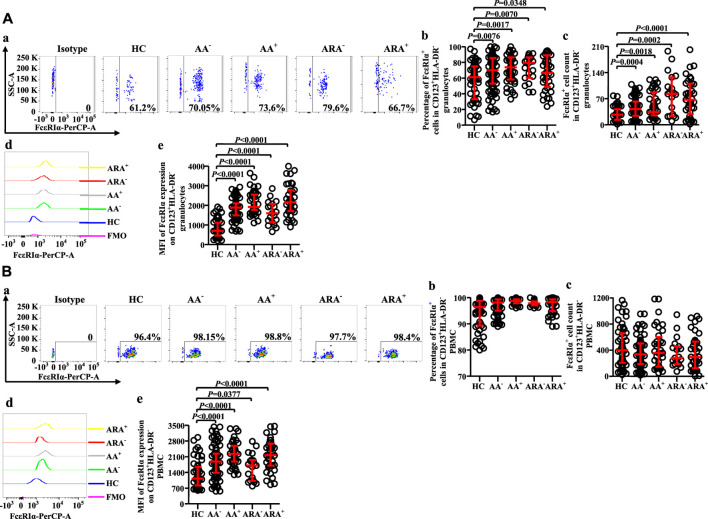
Flow cytometry analysis of FcεRIα expression in CD123+HLA-DR− granulocytes and PBMC of AA and HC subjects. **(A)** and **(B)** proportions and numbers of FcεRIα + cells in CD123+HLA-DR− granulocytes and PBMC. **(a)** Representative graphs of the gating strategies. **(b)** percentages indicated cells of skin prick test (SPT)-negative AA (AA−), SPT-positive AA (AA+), SPT-negative AA combined with allergic rhinitis (ARA−), and SPT-positive AA combined with allergic rhinitis (ARA+) patients and HC subjects. **(c)** numbers of FcεRIα + cells in CD123+HLA-DR− cells indicated. **(d)** representative graphs and **(e)** MFI of FcεRIα expression on CD123+HLA-DR− cells indicated.

FcεRI has long been accepted as a receptor of IgE on basophils, which can mediate allergen-induced basophil activation (Zellweger et al., 2017). We therefore investigated FcεRI expression on CD123^+^HLA-DR^−^ cells (Figures 5A,B). The results showed that the proportions of FcεRI^+^ cells in CD123^+^HLA-DR^−^ granulocytes of AA and ARA patients markedly increased (Figure 5Aa, b, MWUT, *p* = 0.0076, 0.0017, 0.0070, 0.0348), and the numbers of FcεRI^+^ cells in CD123^+^HLA-DR^−^ granulocytes elevated by 56.2% and 24.3% of AA^−^ and AA^+^ patients and 2.1 and 1.5 folds of ARA^−^ and ARA^+^ patients (Figure 5Ac, MWUT, *p* = 0.0004, 0.0018, 0.0002, <0.0001). MFI of FcεRI on CD123^+^HLA-DR^−^ granulocytes of AA and ARA patients also markedly increased (Figure 5Ad, e, MWUT, *p* < 0.0001, 0.0001, 0.0001, 0.0001). Likewise, the MFI of FcεRI on CD123^+^HLA-DR^−^ PBMC of AA and ARA patients increased (Figure 5Bd, e, MWUT, *p* < 0.0001, *p* = 0.0001, 0.0377, 0.0001).

### Correlations between CD63, CD203c, and FcεRIα expression in granulocytes and peripheral blood mononuclear cell of allergic asthma and allergic rhinitis

**FIGURE 6:**
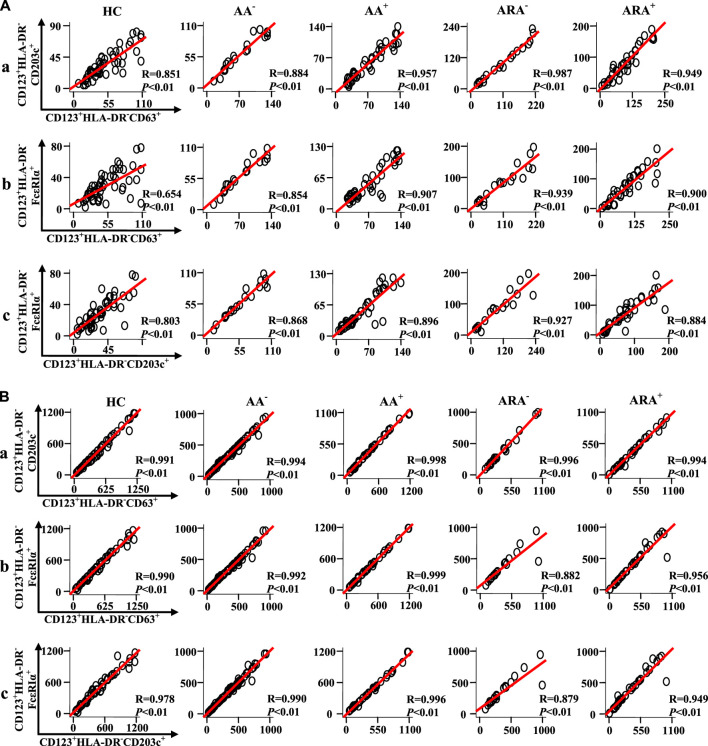
Scatter plots, linear regression lines, and coefficients between the variables of patients with allergic rhinitis (AR) and HC subjects. Correlations between numbers of CD63 +, CD203c +, and FcεRIα + cells in CD123+HLA-DR− granulocytes **(A)** and PBMC **(B)** of skin prick test (SPT)-negative AA (AA−), SPT-positive AA (AA+), SPT-negative AA combined with allergic rhinitis (ARA−), SPT-positive AA combined with allergic rhinitis (ARA+) patients and HC subjects were analyzed using Pearson’s correlation test. Indicated cells were counted in 100,000 leukocytes. Correlation coefficient ranges were defined as follows: *R* < 0.3 as a weak correlation, 0.3 ≤ *R* ≤ 0.7 as a moderate correlation, and *R* > 0.7 as a strong correlation. *p* < 0.05 was taken as statistically significant.

To learn more about the relationships between expressions of CD63, CD203c, and FcεRIα in CD123^+^HLA-DR^−^ granulocytes and PBMC of AA and ARA patients, Pearson’s correlation test was employed. It was observed that there were strong correlations between the numbers of CD63^+^CD123^+^HLA-DR^−^, CD203c^+^CD123^+^HLA-DR^−^, and FcεRIα^+^CD123^+^HLA-DR^−^ granulocytes (Figure 6Aa, b, c Pearson’s correlation analysis, *p* < 0.01 for all), and PBMC (Figure 6Ba, b, c, Pearson’s correlation analysis, *p* < 0.01 for all) of HC, AA, and ARA patients besides CD63^+^CD123^+^HLA-DR^−^ and FcεRIα^+^CD123^+^HLA-DR^−^ granulocytes of HC subjects (Figure 6Ab, Pearson’s correlation analysis, *p* < 0.01 for all).

### Flow cytometry analysis of expressions of CD123, CD63, CD203c, and FcεRIa in KU812 cells

**FIGURE 7:**
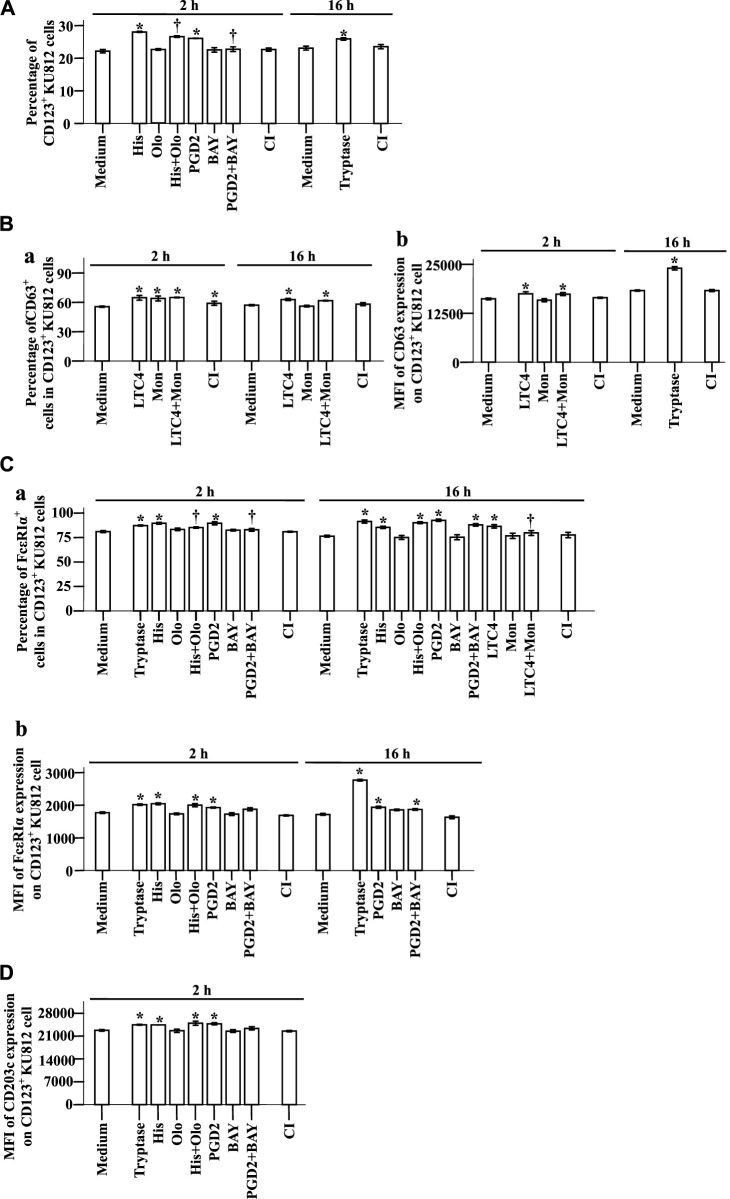
Expression of CD123, CD63, CD203c, and FcεRIα on KU812 cells. The cells were incubated with tryptase (1.0 μg/ml), histamine (1.0 μg/ml), olopatadine hydrochloride (Olo, 10.0 μg/ml), prostaglandin D2 (PGD2, 0.1 μg/ml), BAY-u-3405 Ramatroban (BAY, 0.35 μg/ml), leukotriene C4 (LTC4, 300 nM), montelukast sodium (Mon, 900 nM), calcium ionophore (CI, 1.0 μM), or PBS (pH 7.4) for 2 or 16 h at 37°C. **(A)** percentages of CD123+ KU812 cells at 2 and 16 h following incubation. **(B)** and **(C)** CD63 and FcεRIα expression on CD123+ KU812 cells. **(a)** percentages of CD63+ or FcεRIα cells in CD123+ KU812 cells and **(b)** MFI of CD63 or FcεRIα expression on CD123+ KU812 cells at 2 and 16 h following incubation. **(D)** MFI of CD203c expression on CD123+ KU812 cell at 2 and 16 h following incubation. The data are presented as the mean ± SE for 4 separate experiments. *p < 0.05 compared with a medium alone group, ^†^ *p* < 0.05 compared with the stimulus alone group.

To understand the potential mechanisms of the elevated expression of CD123, CD63, CD203c, and FcεRIa in basophils, we investigated the effect of the proinflammatory mediators on KU812 cells. The results showed that histamine and PGD2 at 2 h following incubation enhanced proportions of CD123^+^ KU812 cells, which were diminished by histamine H1 receptor blocker Olo (Sharif et al., 1996) and PGD2 receptor antagonist BAY (Ishizuka et al., 2004), respectively (Figure 7A, *t*-test, *p* = 0.0002, 0.0056, 0.0108, 0.0092). LTC4 elevated CD63^+^ cells in CD123^+^ KU812 cells at 2 and 16 h (Figure 7Ba, *t*-test, *p* = 0.0001, 0.0030) and the MFI of CD63 expression on CD123^+^ KU812 cell at 2 h following challenge (Figure 7Bb, *t*-test, *p* = 0.0309). LTC4 also increased the proportion of FcεRI^+^CD123^+^ KU812 cells at 16 h, which was clearly reduced by Mon, an LTC4 receptor antagonist (Meshram et al., 2020) (Figure 7Ca, *t*-test, *p* = 0.0006, 0.0421). Likewise, histamine and PGD2 at 2 and 16 h following incubation enhanced proportions of FcεRI^+^CD123^+^ KU812 cells (Figure 7Ca, *t*-test, *p* = 0.0040, 0.0045, 0.0013, 0.0003) and MFI of FcεRI expression on CD123^+^ KU812 cell at 2 h following challenge (Figure 7Cb, *t*-test, *p* = 0.0006, 0.0258). Olo and BAY inhibited histamine and PGD2 induced an increase in FcεRI^+^CD123^+^ KU812 cells at 2 h, respectively (Figure 7Ca, *t*-test, *p* = 0.0216, 0.0342). Histamine and PGD2 also provoked the upregulation of MFI of CD203c expression on CD123^+^ KU812 cell at 2 h following challenge (Figure 7D, *t*-test, *p* = 0.0075, 0.0025). Tryptase increased proportion of CD123^+^ KU812 cells (Figure 7A, *t*-test, *p* < 0.0390) and MFI of CD63 expression on CD123^+^ KU812 cell at 16 h (Figure 7Bb, *p* < 0.0001), proportion of FcεRI^+^CD123^+^ KU812 cells (Figure 7Ca, *t*-test, *p* < 0.0001) and MFI of FcεRI expression on CD123^+^ KU812 cell at 2 and 16 h (Figure 7Cb *t*-test, *p* = 0.0013, <0.0001), and MFI of CD203c expression on CD123^+^ KU812 cell at 2 h (Figure 7D *t*-test, *p* = 0.0067) following challenge.

### Expression of CD63 on blood basophils of allergic mice

**FIGURE 8:**
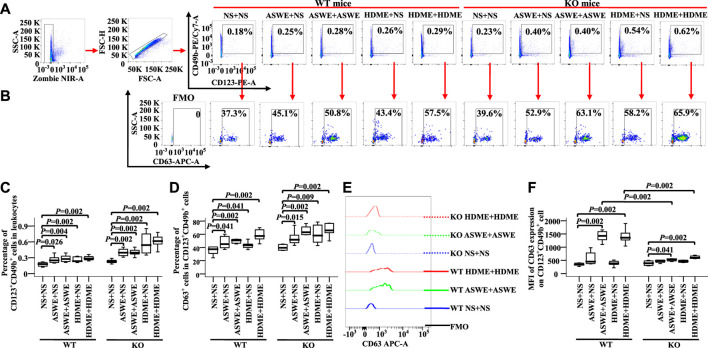
Expression of CD63 in blood basophils of wild-type (WT) and FcεRIα knockout (KO) mice. **(A)** and **(B)** gating strategies and representative figures of CD49b + CD123+basophils in leukocytes and the expression of CD63 in basophils. **(C)** percentages of CD49b + CD123+ basophils in leukocytes. **(D)** percentages of CD63 + cells in CD49b + CD123+basophils. **(E)** representative graph of MFI of CD63 expression in CD49b + CD123+basophil and **(F)** MFI of CD63 expression in CD49b +CD123+basophil. Data were displayed as boxplots for ASWE-sensitized and ASWE-challenged (ASWE + ASWE), ASWE-sensitized and normal saline challenged (ASWE + NS), HDME-sensitized and HDME-challenged (HDME + HDME), HDME-sensitized and normal saline challenged (HDME + NS), and NS control mice (NS + NS), which indicated the median, interquartile range, and the largest and smallest values for the number of experiments indicated. A total of seven animals were in each group. FMO = fluorescence minus one control. *p < 0.05 was taken as statistically significant.

To confirm the effect of allergens on basophils of AA *in vivo*, we investigated CD63 expression on basophils of AA mice sensitized and challenged by allergen extracts ASWE and HDME. The results showed that ASWE + NS, ASWE + ASWE, HDME + NS, and HDME + HDME induced increase in percentages of blood basophils (Figures 8A,C, MWUT, *p* = 0.026, 0.004, 0.002, 0.002, 0.002, 0.002, 0.002, and 0.002) and CD63^+^ basophils (Figures 8B,D, MWUT, *p* = 0.041, 0.002, 0.041, 0.002, 0.015, 0.002, 0.009, and 0.002) in WT-AA mice and FcεRI-KO AA mice. Both ASWE + ASWE and HDME + HDME provoked the enhanced MFI of CD63 expression on basophils of WT-AR mice and FcεRI-KO AR mice (Figures 8E,F MWUT, *p* = 0.002, 0.002, 0.041, and 0.002). However, enhanced MFI of CD63 expression on basophils of WT-AR mice appeared more than that of FcεRI-KO AR mice (Figures 8E,F MWUT, *p* = 0.002, 0.002).

## Discussion

The markedly increased CD123^+^HLA-DR^−^ cells in granulocytes of AA^−^, AA^+^, ARA^−^, and ARA^+^ patients implicate that this cell population may be related to the development of AA and ARA. However, the density of CD123 expression on a single CD123^+^HLA-DR^−^ granulocyte of AA^−^, AA^+^, ARA^−^, and ARA^+^ patients remain unchanged. Taking increased CD123 expressing granulocytes and unchanged density of CD123 expression on a single CD123^+^HLA-DR^−^ granulocyte together, our data support that CD123 is a stable marker on CD123 expressing granulocytes as previously reported that CD123 behaves as a fixed marker in basophils throughout an activation process (Chirumbolo et al., 2011).

Using a specific antibody against unique cell marker molecules and flow cytometry technique allows the identification of previously unknown cells. However, the morphology of cells cannot be seen by this technique. To confirm the cell types that express CD123 in peripheral blood granulocytes and PBMC, we isolated blood CD123^+^HLA-DR^−^ cells by cell sorting technique and examined them under the microscope. We demonstrated for the first time that CD123^+^ granulocytes are mainly composed of basophils, eosinophils, and neutrophils and therefore changes in CD123^+^ granulocytes should result from the alteration of any of them. As previously reported, we have shown that CD123^+^HLA-DR^−^ cells in PBMC are almost all basophils regardless they express CD203c, CD63, or FcεRIα. In the present study, CD123^+^HLA-DR^−^ granulocytes seemed to include basophils (CD203c^+^CD123^+^HLA-DR^−^ cells and some of FcεRIα^+^CD123^+^HLA-DR^−^ cells) as CD203c most likely exclusively expressed by basophils (B uhring et al., 2001) and all basophils express FcεRIα (Riske et al., 1991), a group of eosinophils (FcεRIα expressing eosinophils) (Gounni et al., 1994), and a subpopulation of neutrophils (CD123^+^HLA-DR^−^ cells). Nevertheless, further detailed work is required to confirm these issues. The increased CD123^+^ eosinophils not only confirm that eosinophils can express CD123 but also prove that CD123^+^ eosinophils, most likely mature eosinophils, are markedly elevated in the blood of AA and ARA patients. We also confirm that almost all CD123^+^HLA-DR^−^ PBMC are basophils and therefore changes in the proportions, numbers, and MFIs of these cells confidently represent basophils. The number of CD123^+^HLA-DR^−^ PBMC and MFI of CD123 expression on PBMC had little change in the blood of AA and ARA patients in comparison with HC subjects, suggesting that CD123^+^ basophils in PBMC may not be involved in the development of AA and ARA or could be directly recruited from the blood circulation to the airway lumen (Santos et al., 2016). A report that a decreased percentage of CD123^+^ basophils was observed in the blood after the allergen challenge of the asthmatic lung (Santos et al., 2016) may help explain our observation.

CD203c and CD63 have been recognized as activation markers of basophils. The CD203c activation pattern is characterized by a rapid and significant upregulation, reaching maximum level after 5–15 min of stimulation. In the CD63 activation pattern, maximum upregulation of CD63 was detected only after 20–40 min, and upregulation reached a maximum level after 60 min. These two activation patterns are linked to two different mechanisms of basophil activation (Hennersdorf et al., 2005). Increased expression of CD203c, but not CD63 on basophils, is accompanied by asthma exacerbation (Ono et al., 2010). We find that almost all CD123^+^HLA-DR^−^ cells expressed CD63 regardless of whether they were in granulocyte or PBMC populations and HC, AA, or ARA subjects. The enhanced expressions of CD63 in CD123^+^HLA-DR^−^ granulocytes and PBMC implicate that these cells, possibly including basophils, eosinophils, and neutrophils are activated in AA and ARA as CD63 has been shown to express on neutrophils (Kinhult et al., 2002), eosinophils (Mahmudi-Azer et al., 2002), and T cells (Pfistershammer et al., 2004) apart from basophils. Likewise, increased CD203c expression on granulocytes and PBMC indicate that activated basophils are elevated in AA and ARA patients because CD203c is a selective marker of basophil activation (Kim et al., 2016). Based on these results, we believe that both CD63 and CD203c are sensitive enough as activation markers, but CD203c seems more selective for basophils.

As for CD63 and CD203c, the enhanced expression of FcεRI^+^ cells is found in CD123^+^ granulocytes. These enhanced FcεRI expressing cells in the granulocyte population should be eosinophils as only approximately 32% purified eosinophils express FcεRI (Gounni et al., 1994) and no FcεRI negative basophils have been reported (Berings et al., 2018); neutrophils do not express FcεRI (Smith et al., 1995). The strong correlations between numbers of CD63^+^CD123^+^HLA-DR^−^, CD203c^+^CD123^+^HLA-DR^−^, and FcεRIα^+^CD123^+^HLA-DR^−^ granulocytes and PBMC of HC, AA, and ARA patients implicate that these correlated cells groups may originate from similar or same cell populations.

Although it is not easy for allergens to directly contact inflammatory cells in the body, it is possible that allergens directly act on these cells during allergic inflammation. In the present study, we observed that ASWE enhanced the numbers of CD123^+^HLA-DR^−^ granulocytes and PBMC, and the numbers of CD63^+^CD123^+^HLA-DR^−^ granulocytes and PBMC of AA and ARA patients when it was added to blood for 30 min. ASWE also increased the numbers of CD203c^+^CD123^+^HLA-DR^−^ PBMC and FcεRI^+^CD123^+^HLA-DR^−^ PBMC and the MFI of CD203c expression on CD123^+^HLA-DR^−^ granulocyte of AA and ARA patients. On the other hand, HDME increased the proportion of CD63^+^ cells in CD123^+^HLA-DR^−^ granulocytes and the MFIs of CD63 expression on CD123^+^HLA-DR^−^ granulocytes and PBMC of AA and ARA patients. HDME also elevated the MFI of CD203c expression on CD123^+^HLA-DR^−^ granulocytes and the PBMC of ARA patients. These observations indicate that allergens can alter the behavior of CD123^+^HLA-DR^−^ granulocytes and PBMC, and CD123^+^HLA-DR^−^ granulocytes and PBMC respond to ASWE and HDME differently when these two allergens are directly added to the cells.

It is rather difficult to obtain a large number of pure basophils, we therefore use a basophil cell line KU812 cells instead (Rasheed et al., 2009) to examine the influence of proinflammatory mediators on basophils. We found that histamine, tryptase and PGD2 enhanced proportions of CD123^+^ KU812 cells and FcεRI^+^CD123^+^ KU812 cells, and MFI of FcεRI and CD203c expressions on CD123^+^ KU812 cells. Likewise, LTC4 increased the proportion of FcεRI^+^CD123^+^ KU812 cells and LTC4 and tryptase elevated CD63^+^CD123^+^ KU812 cells. These observations implicate that proinflammatory mediators can modulate basophil surface marker expression, particularly FcεRI expression.

We investigated for the first time the CD63 expression on basophils of ASWE- and HDME-induced AA mice, particularly FcεRI-KO AA mice. The results that ASWE + NS, ASWE + ASWE, HDME + NS, and HDME + HDME all induced increase in percentages of blood basophils, and CD63^+^ basophils in WT-AA mice and FcεRI-KO AA mice are unexpected, but it may suggest that the actions of ASWE and HDME in numbers of basophils may not through a FcεRI-dependent mechanism. However, enhanced MFI of CD63 expression on basophils of WT-AR mice appeared more than that of FcεRI-KO AR mice, implicating that ASWE- and HDME-induced upregulation of CD63 expression may be *via* a FcεRI-dependent mechanism.

In conclusion, upregulated expressions of CD123, CD203c, CD63, and FcεRIα in PBMC and granulocytes of patients with AA and ARA suggest that CD123^+^HLA-DR^−^ cells may contribute to the development of AA and ARA. Modulation of expressions of CD123, CD203c, CD63, and FcεRIα by allergens and proinflammatory mediators implies that allergens may participate in AA and ARA through CD123^+^HLA-DR^−^ cells.

## Data Availability

The original contributions presented in the study are included in the article/Supplementary Material; further inquiries can be directed to the corresponding author.
