# Supplementary material for: Increased expressions of CD123, CD63, CD203c, and Fc epsilon receptor I on blood leukocytes of allergic asthma
Source: Front Mol Biosci. 2022 Aug 11;9:907092. doi: 10.3389/fmolb.2022.907092 (PMC9403487; doi:10.3389/fmolb.2022.907092)
Supplement: Supplementary file 1 [file DataSheet1.pdf]

Supplementary file

Increased number of CD123$^+$HLA-DR$^-$ cells and expressions of CD63, CD203c and FcεRI on blood granulocytes and PBMC of allergic asthma

Hua Xie[1], Liping Chen[4], Huiyun Zhang[3], Junling Wang[2], Yanyan Zang[2], Mengmeng Zhan[3], Fangqiu Gu[2], Shunlan Wang[5], Shaoheng He[1,2]*

[1]The PLA Center of Respiratory and Allergic Disease Diagnosing Management, General Hospital of Northern Theater Command, Shenyang 110016, China;

[2]Allergy and Clinical Immunology Research Centre, the First Affiliated Hospital of Jinzhou Medical University, Jinzhou, Liaoning 12100, China;

[3]Translational Medicine Institute, Shenyang Medical College, Shenyang, Liaoning 110034, China;

[4]Department of Respiratory and Critical Care Medicine, the Second Hospital of Senyang Medical College, Shenyang, Liaoning 110002, China;

[5]Central Laboratory, Affiliated Haikou Hospital of Xiangya Medical College, Central South University, Haikou, Hainan, 570208, China.

Short title: basophil and asthma

***Correspondence to:**

Professor Shaoheng He

Allergy and Clinical Immunology Research Centre, Jinzhou Medical University, No. 2, Section 5, Renmin Street, Guta District, Jinzhou, Liaoning 121001, People's Republic of China.

Tel: 86-416-4605081, Fax: 86-416-4605082.

E-mail: shoahenghe@126.com

FIGURE S1

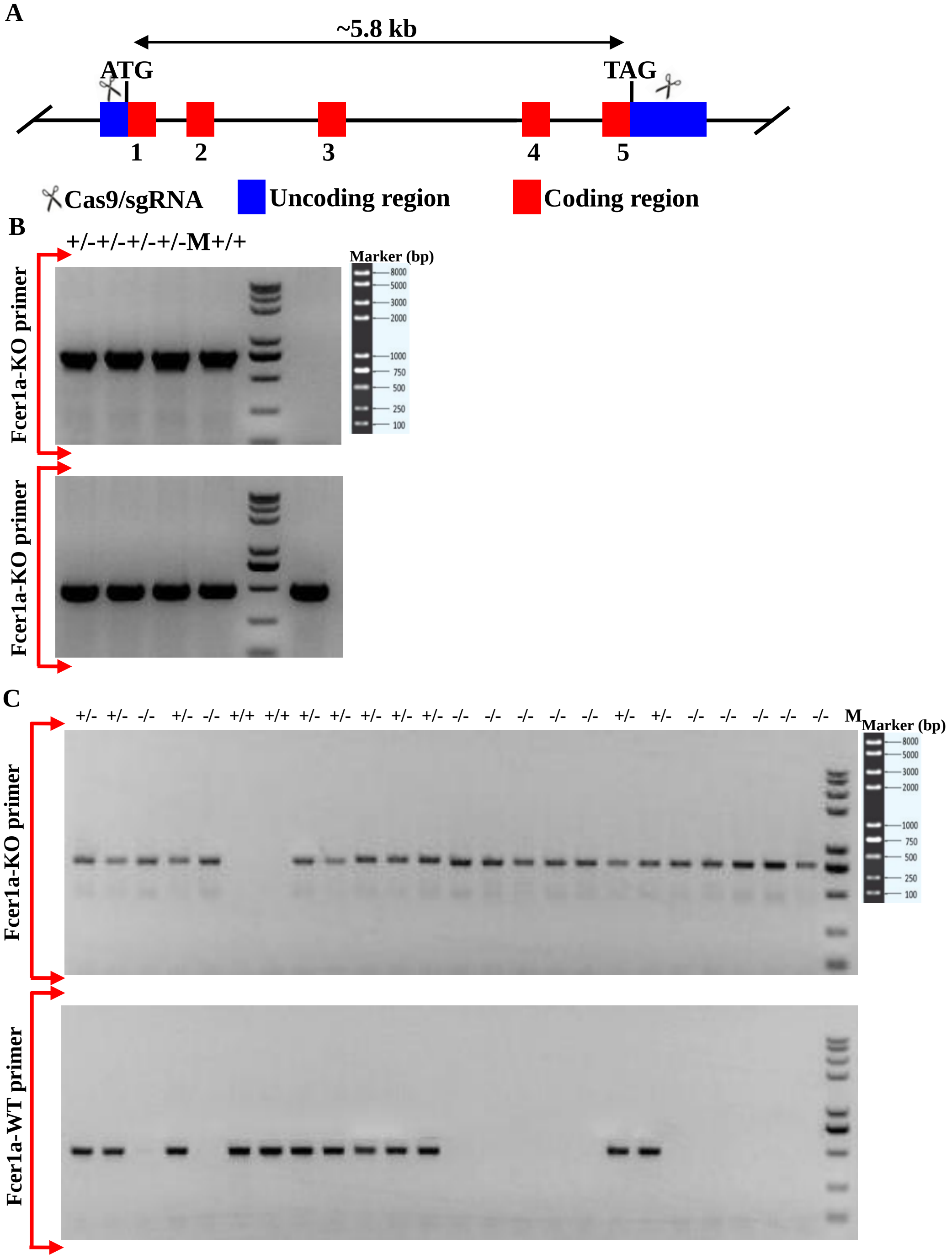

FIGURE S1 CRISPR/Cas9-mediated generation of FcεRIα knockout (KO) mouse model. (**A**) represents view of the CRISPR/Cas9 targeting strategy used for generating FcεRIαKO mice. The schematic sequences of wild-type (WT) alleles are shown. Coding regions are indicated with red boxes and uncoding regions are noted by blue boxes. The conventional start codon (ATG) and termination codon (TAG) are indicated; (**B**) shows representative PCR genotyping results of FcεRIα WT (+/+), and

heterozygous (+/-) F0 mice; (**C**) indicates representative PCR genotyping results of FcεRIα WT (+/+), and heterozygous (+/-) and homozygous (-/-) F2 mice.

FIGURE S2

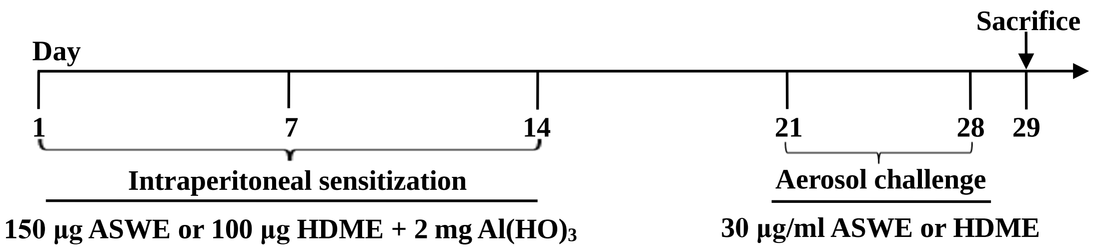

FIGURE S2 The schematic diagram of mouse AA model establishment induced by *Artemisia sieversiana* wild allergen (ASWE) and house dust mite extract (HDME).
